# Treatment by glyphosate-based herbicide alters life history parameters of the rose-grain aphid *Metopolophium dirhodum*

**DOI:** 10.1038/srep27801

**Published:** 2016-06-15

**Authors:** Pavel Saska, Jiří Skuhrovec, Jan Lukáš, Hsin Chi, Shu-Jen Tuan, Alois Honěk

**Affiliations:** 1 Crop Research Institute, Group Functional Diversity of Invertebrates and Plants in Agro-Ecosystems, Drnovská 507, Prague 6 – Ruzyně, 161 06 Czech Republic; 2 National Chung Hsing University, Department of Entomology, 250 Kuo-Kuang Rd., Taichung, 40227, Taiwan, Republic of China

## Abstract

Glyphosate is the number one herbicide in the world. We investigated the sub-lethal effects of this herbicide on the aphid *Metopolophium dirhodum* (Walker), using an age-stage, two-sex life table approach. Three concentrations of the herbicide (low - 33.5, medium - 66.9 and high - 133.8 mmol dm^−3^ of active ingredient) and distilled water as the control were used. The LC_50_ of the IPA salt of glyphosate on *M. dirhodum* was equivalent to 174.9 mmol dm^−3^ of the active ingredient (CI_95_: 153.0, 199.0). The population parameters were significantly negatively affected by herbicide application, and this negative effect was progressive with the increasing concentration of the herbicide. A difference of two orders of magnitude existed in the predicted population development of *M. dirhodum* between the high concentration of the herbicide and the control. This is the first study that comprehensively documents such a negative effect on the population of an herbivorous insect.

Pesticides are either chemical or biological substances or agents that kill or slow down the population growth of a pest or unwanted organism. Contrary to biopesticides which are environmentally safe^1^,^2^, pesticides based on chemical substances, including herbicides designed to kill weeds and other unwanted plants, may represent considerable environmental or health risks^3^,^4^, and as such deserve continuous research attention.

As a non-selective herbicide, glyphosate (N-(phosphonomethyl)glycine) has rapidly become the most widely used chemical ingredient with herbicidal activity in the world^5^,^6^. In agricultural settings, its wide adoption have been largely stimulated by the use of genetically modified herbicide tolerant crops in some countries^7^, but glyphosate can be applied as pre-sowing, pre-harvest or as stubble herbicide in conventional crops^8^ or in non-agricultural situations^5^. Plants take up the herbicide solution by the leaves and systemically translocate it to the entire plant via the vascular system^9^. Glyphosate blocks amino acid synthesis and proteosynthesis, and treated plants become dry and die several days after application^9^.

Glyphosate is traditionally considered to be a chemical with relatively low ecological and toxicological side effects in terrestrial ecosystems^4^,^6^,^10^ because the active ingredient inactivates rapidly when it reaches the soil surface^4^,^9^,^11^, but in aquatic environments it is known to be highly toxic^12^ and recently it became suspected to be carcinogenic to humans^13^. Commercial products containing glyphosate seem to be more toxic than the active ingredient alone, possibly due to the surfactants used in the commercial solutions^9^. Glyphosate-based herbicides have demonstrated some fungicidal activity against rusts on glyphosate-resistant wheat^14^. Other studies provide evidence that environmental concerns about glyphosate may be legitimate also in terrestrial ecosystems. For example, earthworms reduced their activity and reproduction dramatically in a study by Gaupp-Berghausen *et al*.^15^; this reduction in earthworm activity led to the accumulation of some nutrients in the soil. Sub-lethal effects occurred in *Chrysoperla externa* (Walker) fed glyphosate-treated prey^16^. The discovery of glyphosate in the body of the land snail *Cornu aspersum* (O.F. Müller) by Druart *et al*.^11^ suggested the ability of this herbicide to enter the food chain. Further eco-toxicological assessment of this substance or products that contain it is therefore needed.

Although the most studied aspect of eco-toxicological research is the assessment of direct mortality caused by the focal substance or product to non-target organisms, the study of sub-lethal effects, i.e., effects on individual organisms that survive exposure, is equally important. Sub-lethal effects may be manifested in many ways, including changes in the population parameters, physiology, behaviour or ecology of a species^17^ and may result not only from direct contact with herbicide but also as a result of feeding on contaminated food.

The impact of the application of glyphosate-based herbicides on communities and populations of insect herbivores, such as aphids, has been studied only scarcely. Herbicide application can affect herbivore populations in various ways. One mechanism of indirect herbicide impact on populations of herbivorous insects originates in the removal of the host plant or in the destruction of the habitat and modification of its structure. For example, Egan *et al*.^18^ documented a decline in populations of *Therioaphis maculata* (Buckton) along the field edges after herbicide (dicamba and 2,4-D) treatment associated with vegetation removal. In contrast, Dewar *et al*.^19^ and Albajes *et al*.^20^ reported that aphids became more abundant in glyphosate-treated plots in herbicide-tolerant crops, which might be a consequence of the increased vigour of crop plants released from competition with weeds^19^ or a reduction in the associational resistance of crop plants to herbivores due to a disrupted crop-weed interaction underground^21^. Other modes of action of herbicides against herbivorous insects include the direct toxicity of the formulation^22^ or the sub-lethal effects of the herbicides caused by the insects feeding on contaminated or modified, less nutritious food^23^. Available studies, however, present mixed results depending on the taxon and the herbicide used^22^,^23^,^24^,^25^,^26^. Wright *et al*.^22^ demonstrated that the cereal aphid *Rhopalosiphum padi* (Linnaeus) survived spraying or feeding on a sucrose solution of glyphosate, whereas this aphid rapidly died if exposed to the other two herbicides. Kjaer & Heimbach^24^ found that the development, growth rate and fecundity of *Sitobion avenae* (Fabricius) on the wheat remained unaltered under recommended field rates of metsulfuron-methyl herbicide, and the survival and relative growth rates were unaffected when *Pieris brassicae* (Linnaeus) and *Gastrophysa polygoni* (Linnaeus) larvae were fed *Brassica napus* Linnaeus or *Fallopia convolvulus* (Linnaeus), respectively, treated with sulfonylurea herbicides. Burrows *et al*.^25^ did not find any effect of herbicide treatment on the colonization and population development of *Aphis glycines* Matsumura in a glyphosate-tolerant soybean crop. In contrast, Lipok^26^ reported that *Aphis fabae* Scopoli was repelled by four different herbicidal substances, including glyphosate, which had been freshly sprayed on faba bean plants. In the same study, aphid reproduction was also reduced when aphids were reared on plants sprayed by the field dose of glyphosate, indicating systemic effects of the herbicides on the aphids^26^. Recently, Hahn *et al*.^23^ tested two herbicides for possible sub-lethal effects; they applied glyphosate-based Roundup LB Plus and sulfonylurea-based Atlantis AG to three species of host plants to *Mamestra brassicae* (Linnaeus) caterpillars. Of the six combinations, only one combination caused a significantly negative effect on the caterpillar development, and none of the combinations that included glyphosate caused any harm to the caterpillar development^23^. Therefore, the available literature does not provide sufficient data for understanding the response of insect herbivores to herbicide use in agro-ecosystems. This can only be achieved by detailed study on the demography of herbivorous insects after herbicide application^27^,^28^.

The aim of this study, therefore, was to study comprehensively and for the first time how treatment with glyphosate-based herbicide affects the demography and population development of a fluid-feeding herbivore by using the cereal aphid *Metopolophium dirhodum* (Walker) (Sternorrhyncha: Aphididae) and wheat *Triticum aestivum* Linnaeus as the herbivore-plant system. We adopted the age-stage, two-sex life table^29^,^30^ for estimating the population parameters and development. This theory considers both sexes and the variable developmental rate among individuals and can properly describe the development, stage differentiation, survival, and the reproduction of the population. Age-stage, two-sex life table can (and should) also be applied for parthenogenetic populations because contrary to the traditional female-based life table, it takes the pre-adult development and mortality into account and thus provides more accurate estimates of population parameters^31^,^32^. This approach has been successfully used for the assessment of the sub-lethal effects of glyphosate-based herbicides on the population parameters of the predatory lacewing *Ch. externa*^16^, which was fed prey treated with glyphosate-based herbicide. The fecundity, fertility and all major population parameters were greatly affected by herbicide contamination of the food in *Ch. externa*^16^. As the aphids will be in direct contact with the herbicide, we hypothesize that population parameters of *M. dirhodum* will be negatively affected by the herbicide treatment.

## Results

**Figure 1:**
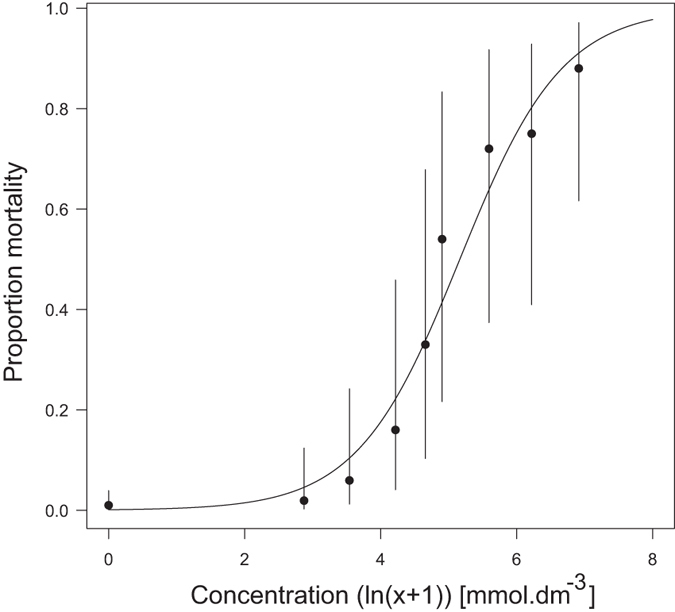
Observed proportion of *Metopolophium dirhodum* that died as a result of the application of glyphosate-based herbicide. The line represents the modelled response of the proportion mortality to increasing herbicide concentration based on the following logistic regression: logit (Proportion mortality) = −6.8515 + 1.3267 ln (Concentration + 1) [mmol dm^−3^ of the a.i.].

The mortality of the aphids sprayed with the distilled water in the toxicity test was low after 24 h (2 individuals out of 400), suggesting that the method of spraying is not detrimental to the aphids. The mortality of *M. dirhodum* increased with the increasing concentration of herbicide (Fig. 1). Based on the results of the toxicity test of eight concentrations of the herbicide on *M. dirhodum* (see the Material and Methods section), the estimated values of the LC_50_ and LC_90_ were 174.9 mmol dm^−3^ of the a.i. (CI_95_: 153.0, 199.0) and 916.6 mmol dm^−3^ of the a.i. (CI_95_: 694.5, 1209.6), respectively. Based on this model, the mortality was estimated for the three concentrations of glyphosate-based herbicide used in the life table experiments: 10.4% (low), 22.2% (medium) and 33.8% (high concentration).

**Figure 2:**
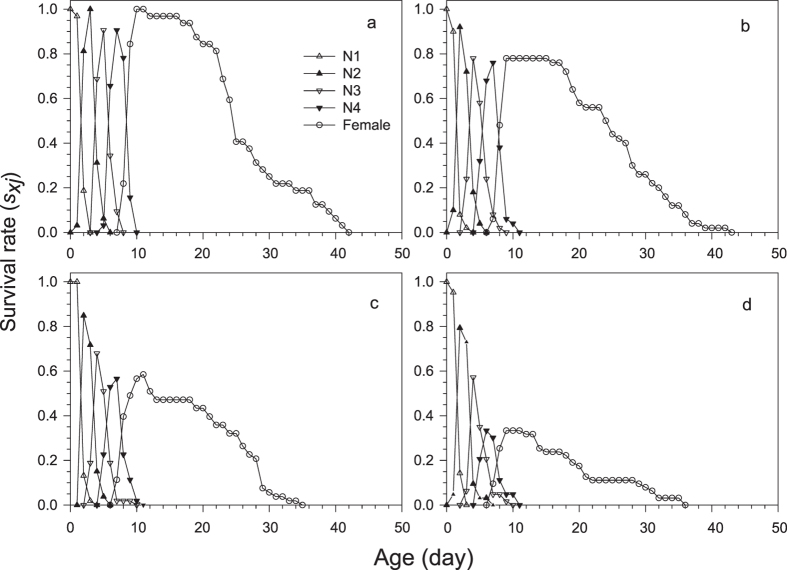
Age-stage-specific survival rates (*s*_*xj*_) of *Metopolophium dirhodum* whose parental females were treated by glyphosate-based herbicide. (**a**) – control, (**b**) – low concentration, (**c**) – medium concentration, (**d**) – high concentration.

**Figure 3:**
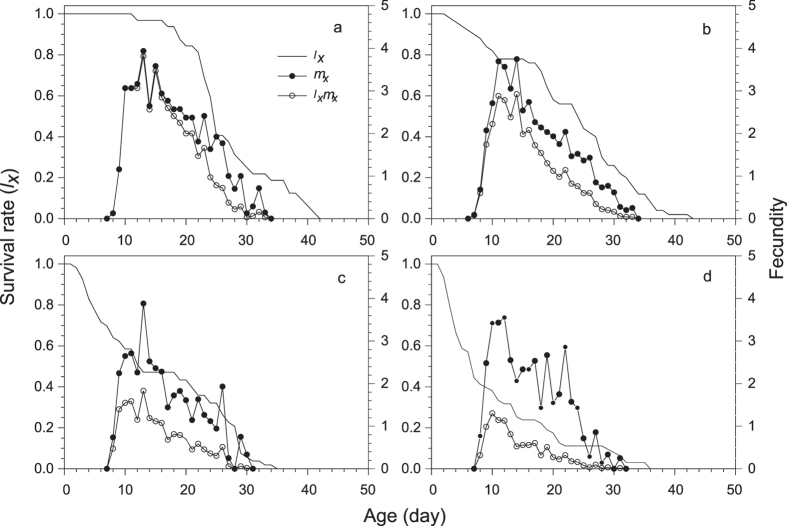
Age-specific survival rates (*l*_*x*_), age-specific fecundity (*m*_*x*_), and net maternity (*l*_x_*m*_*x*_) of *Metopolophium dirhodum* whose parental females were treated with glyphosate-based herbicide. (**a**) – control, (**b**) – low concentration, (**c**) – medium concentration, (**d**) – high concentration.

**Figure 4:**
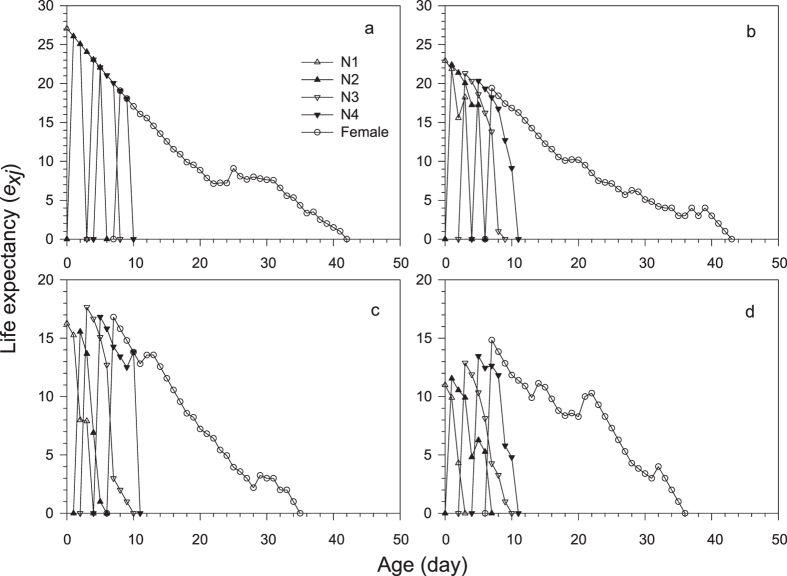
Age-stage-specific life expectancy (*e*_*xj*_) of *Metopolophium dirhodum* whose parental females were treated with glyphosate-based herbicide. (**a**) – control, (**b**) – low concentration, (**c**) – medium concentration, (**d**) – high concentration.

**Figure 5:**
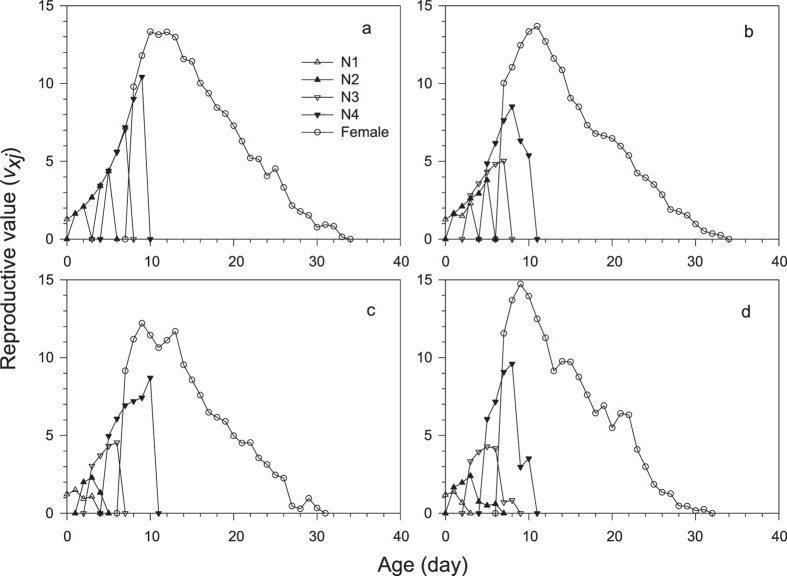
Age-stage-specific reproductive value (*v*_*xj*_) of *Metopolophium dirhodum* whose parental females were treated with glyphosate-based herbicide. (**a**) – control, (**b**) – low concentration, (**c**) – medium concentration, (**d**) – high concentration.

The development of filial *M. dirhodum* was rapid in all treatments and with negligible variation among the treatments in Experiment 1 (Table 1). The age-stage specific survival rates (*s*_*xj*_) demonstrate that due to variation in duration of development of particular stages within cohorts, as many as three different stages were present at a time (Fig. 2). The average duration of the adult stage varied across treatments and tended to decrease with herbicide concentration (Table 2), which, together with the pre-adult survival that continuously decreased with herbicide concentration, caused that the average longevity to significantly decrease with each increase in the herbicide concentration (Table 2). The reproduction parameters were also affected by herbicide treatments; however, the lowest concentration of herbicide did not show any indication of negative effects, and the duration of the pre-oviposition period was even slightly shorter than for the control (Table 2). The age-specific survival rates (*l*_*x*_), age-specific fecundity (*m*_*x*_) and net maternity (*l*_*x*_*m*_*x*_) for each treatment shown in Fig. 3 and the age-stage-specific life expectancy (*e*_*xj*_) in Fig. 4 together demonstrate the overall impact of the increase in herbicide concentration on the population of *M. dirhodum* over time. The values of the estimated intrinsic rate of increase, *r*, and finite rate, *λ*, indicated a rapid population growth of *M. dirhodum* in all treatments (Table 2); nevertheless, the application of herbicide significantly negatively affected the population parameters of this aphid species. The magnitude of this effect varied among parameters. While each increase in herbicide concentration caused a significant decrease in *R*_*0*_, the *T* decreased regardless of the herbicide treatment compared to the control (Table 2). Both parameters of population growth, *r* and *λ*, were unaffected by the lowest concentration and similarly decreased due to the medium and high concentrations of herbicide (Table 2). The highest age-stage reproductive value (*v*_*xj*_) was estimated to occur at a similar age (9–11 days) in all treatments (Fig. 5). The long-term sub-lethal effects of the herbicide application on the population biology thus occur prominently in the filial generation of the aphid *M. dirhodum*.

The fecundity, oviposition days and duration of adult stage were also estimated for the treated females in Experiment 2 (Table 2). As in Experiment 1, a negative effect of the herbicide treatment occurred on these population parameters (Table 2). In the case of fecundity and female stage duration, the decrease in the observed values after herbicide application seemed to be more obvious in the parental generation (Experiment 2) than in the filial generation (Experiment 1) (Table 2). With the exception of oviposition days in the control and the high herbicide concentration, the values of all parameters were significantly lower in Experiment 2 compared to Experiment 1 (Table 2).

**Figure 6:**
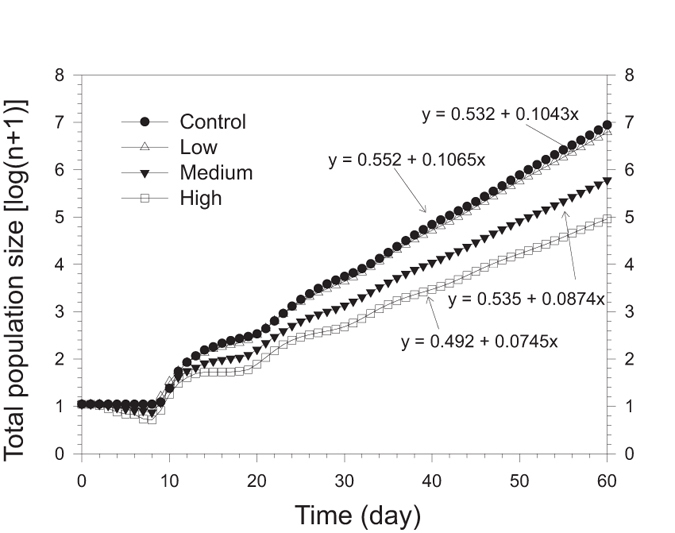
Comparison of population projections for *Metopolophium dirhodum* treated with three concentrations of glyphosate-based herbicide or as the control, based on the age-stage, two-sex life table. The regression formulas describe the linear population growth of each cohort from day 40 onwards as the population approached the stable age-stage distribution.

The population projection based on the age-stage, two-sex life table using the data from Experiment 1 shows that treatment with the glyphosate-based herbicide affects the population growth of *M. dirhodum* (Fig. 6). According to the simulation, the difference in population size after 60 days will differ by two orders of magnitude and will reach approximately 8.76 million aphids with the control, 6.20 million at the low concentration, 0.60 million at the medium concentration and 0.09 million at the high concentration of the herbicide. The population growth curves (in a logarithmic scale) approach linearity after approximately 40 days (Fig. 4), which suggests that aphid populations approached the stable age-stage distribution. The slopes of the regression lines that describe such linear population increase are equal to log(*λ*) for each cohort.

## Discussion

The main objective of this paper was to investigate the sub-lethal effects of a glyphosate-based herbicide on the population development of the cereal aphid *Metopolophium dirhodum*. Based on an age-stage, two-sex life table analysis, the acquired data provide evidence that this aphid species is sensitive to the doses (when applied by spraying) recommended by a producer of Roundup Aktiv, a glyphosate-based herbicide commercially available in the Czech Republic. All life table parameters of the filial generation, *r, λ, R*_*0*_ and *T*, as well as the fecundity, duration of oviposition period, longevity and survival of both the treated and filial generations, were negatively and progressively affected by increasing the concentration of the glyphosate-based herbicide. As such, this is the first report that comprehensively shows the negative effect of a glyphosate-based herbicide (or any herbicide) on the life table parameters of an herbivorous insect. The life table approach has not been used until now for the ecotoxicological assessment of the sub-lethal effects of herbicides on herbivorous insects, which is indeed surprising.

As a result of the variation in the life table and population parameters, the population growth of *M. dirhodum* varied enormously among the treatments. In the control treatment, the aphid population was predicted to grow to reach a population size of 8.76 million in 60 days in the absence of diseases, predators or parasitoids and with unlimited resources, whereas in the case of the high herbicide concentration, the population size was predicted to be only 91,000 aphids. This difference is in the order of two magnitudes. The impact of the glyphosate treatment on the population development of *M. dirhodum* is enormous.

Herbicides are primarily designed to kill weeds. The power to suppress other noxious organisms such as aphids (this paper) or rusts^14^ may be perceived as desirable from the pest control perspective. But not all herbivorous insect species that occur on herbicide-managed sites are pests, and many of them deserve protection. Here, in addition to showing that direct mortality occurs when applying high doses of glyphosate-based herbicide (the LC_50_ for *M. dirhodum* estimated in this study is higher than the field-recommended concentration by the manufacturer^33^), the life table parameters of an herbivore were also changed when using the recommended concentrations. Individual aphids that survive the treatment and leave the treated and destroyed environment thus suffer a reduced ability to reproduce, which reduces the chance to build up new populations on reaching a refuge. This newly ascertained negative effect of an herbicide on the life table parameters of an herbivorous insect must, therefore, be seen as a potential threat to the biodiversity of the herbivorous insects on conventionally managed arable land and public green spaces, i.e., in areas where herbicides are predominantly used^5^.

Another aspect of the existence of sub-lethal effects on herbivorous insects that deserves attention is the position of herbivorous insects in the food web. Feeding on herbicide-treated prey may be harmful to some insect predators^16^, but how this herbicide treatment-generated decrease in quality of herbivorous insects as food for insect predators and parasitoids occurs over generations is largely unexplored. We simply know too little about the fate of the herbicides in the terrestrial food webs. Studies based on life tables are important to reveal the long-term and overall effect of herbicides at the population level.

The life tables of *M. dirhodum* estimated herein represent a typical aphid life table with high reproduction rate, short generation time, high intrinsic rate of increase and finite rate of increase of the population. Although a number of studies have estimated life tables of aphids using conventional methods, the age-stage, two-sex life table literature is sparse for aphids, and to the best of our knowledge, no life table data have been published for *M. dirhodum* so far. This is perhaps because it feeds exclusively on leaves^34^ and research attention focused mainly on aphids feeding on ears of cereals. Detection of the stage differentiation and stage structure in parthenogenetic populations is, however, also important. Data for other species of aphids have to be used for comparison, among which *S. avenae*, the species that feeds on ears of cereals, has been the most studied^35^,^36^,^37^,^38^. In general, the life table parameters estimated in this study are somewhat similar to the published data on aphids^31^,^35^,^36^,^37^,^38^,^39^,^40^,^41^,^42^,^43^, although the variation in the literature is huge for several reasons. In the first place, a comparison of parameters for different insect species reared on different species of plants may be tricky because the evidence is clear that life table parameters of an herbivore population can be dramatically affected even by lineages or varieties of the same host plant^40^,^41^,^42^. The second problem that limits the use of available literature data for comparison is that thermal conditions differ among studies, and as a consequence, temperature notably influences development^43^ and life table parameters^39^,^44^,^45^. The third problem is that a comparison of life table parameters calculated by using the age-stage, two-sex life table to parameters from the traditional female age-specific life table is inappropriate; Huang and Chi^46^ demonstrated earlier the problems associated with the application of female age-specific life tables to insect populations. The limited literature on life tables of cereal aphids therefore suggests that more investigations using proper methods should be conducted for a better understanding of which conditions are important for population build-up of these important pests in the field.

Importantly, the use of approaches other than life tables might deliver different results and conclusions on the effects of glyphosate-based herbicides. Using life tables, however, provides a more complete answer to the research questions related to the population biology of a species when the sub-lethal effects of pesticides are the focus. We therefore see a strong need for further studies on the sub-lethal effects of herbicides on non-target organisms through the use of age-stage, two-sex life tables. Biological and ecological interactions, even in simplified systems such as agricultural crops, are complex, and an understanding of how pesticide applications modify the roles of the individual components of natural food webs including insect herbivores, such as aphids, would be useful.

## Materials and Methods

### Aphids

*M. dirhodum* was used as the model herbivore species. It is an oligophagous aphid species^47^ that occurs abundantly on leaves of all cereal crops in Central Europe in its summer phase^34^, and is very easy to rear in laboratory cultures. Viviparous wingless or winged females are 2–3 mm long and develop through four larval instars. In this experiment, we used a laboratory strain of *M. dirhodum* that had been maintained for 15 years in the Crop Research Institute, Prague. The aphids were maintained on young (stages 12–13 according to the BBCH scale) plants of winter wheat (*Triticum aestivum* Linnaeus) in a greenhouse with the temperature regulated to approximately 20 ± 1 °C and under a natural photoperiod.

### Herbicide application

Roundup Aktiv (Monsanto, Antwerp, Belgium) was used as the glyphosate-based herbicide in this study. Roundup Aktiv has been approved for use in all kinds of agricultural and horticultural conditions and for non-agricultural environments in the Czech Republic. This herbicide contains 229 g of the isopropylamine (IPA) salt of glyphosate (molecular weight = 228.18 g mol^−1^) as the active ingredient (a.i.) per litre of the solution (1.004 mol dm^−3^)^33^. The herbicide was applied on aphids by means of the auto-load Potter Precision Laboratory Spray Tower (Burkhard Scientific, Uxbridge, UK) with a spraying area of 110 cm^2^. Adult wingless aphids were placed on filter paper in a Petri dish (6 cm in diameter), and the dish with aphids was positioned on the auto-load arm at the centre on the floor of the application chamber of the Potter tower. For treatments, 2 ml of the solution (either distilled water or a solution of herbicide) was sprayed into the application chamber at a pressure of 3 bar.

### Toxicity test

An *a priori* toxicity test of the herbicide on *M. dirhodum* was made to detect the direct mortality within 24 h after the herbicide application. Eight different molar concentrations of the herbicide (diluted in distilled water) were used in this experiment: 16.7, 33.5, 66.9, 104.4, 133.8, 267.6, 501.8 and 1003.8 mmol dm^−3^ of the a.i. and pure distilled water as the control. These concentrations were chosen for the precise identification of the LC_50_ and LC_90_ of *M. dirhodum* after correction for the sprayed area and partial adhesion of the aerosol to the sides of the chamber and were equivalent to 40, 80, 160, 240, 320, 640, 1200 and 2400 ml of the commercial product diluted in 2 l of water applied per 100 m^2^. Ten replicates were performed for each concentration, with each replicate containing 10 aphids. The mortality data were expressed as a binary vector containing the number of surviving and dead aphids per dish and treatment and analysed using GLM logit analysis in the software R version 2.15 48 while assuming a binomial distribution of errors. Molar concentration of the herbicide + 1 (to enable the ln transformation) was used as the explanatory variable. Examination of the residuals confirmed the accuracy of the fit of the models to the data^49^.

### Experimental setup of the life table study

Three different molar concentrations of the herbicide (diluted in distilled water) were used in the life table study: 33.5 (low), 66.9 (medium) and 133.8 mmol dm^−3^ of the a.i. (high) and pure distilled water as the control. These concentrations were chosen according to the recommended dosages in agricultural systems by the manufacturer^33^ after correction for properties of the spraying device (see above) and were equivalent to 80, 160 and 320 ml of the commercial product diluted in 2 l of water applied per 100 m^2^. The highest concentration used in this study represents the maximum recommended concentration doubled, a situation that may occur in the field as a result of overlapping applications. The sprayed aphids were immediately transferred individually to growing pots with 2–3 new plants of winter wheat (stage 11 according to the BBCH scale) isolated within a transparent polyethylene tube and covered from the top by a fine mesh to reduce the risk of escape. Experiment 1 was conducted to study the long-term (i.e., over generations) sub-lethal effect of herbicide application on the life table of *M. dirhodum*, so the life table data were collected for the filial generation. We allowed the treated females to produce nymphs overnight and transferred the newly born nymphs (1–2 per female) to a new wheat plant (stages 10–11 according to the BBCH scale; 80–100 replications per treatment). Each day, the aphid instar was recorded, and after reaching adulthood, the newly born aphids from these adults were counted until death. The born nymphs were removed every day. Data for the winged adults were discarded from the analysis as these tended to escape in abundance from our isolators. Experiment 2 was designed to study the short-term (i.e., in the same generation) sub-lethal effect of herbicide application on fecundity and survival of the treated *M. dirhodum*. A cohort of 23–28 newly hatched apterous females was treated with herbicide as described above and monitored until death. Each day, the offspring were counted and removed. In both experiments, the individuals were transplanted to new wheat plants every three to four days to facilitate manipulation. The individuals included in this experiment were not mothers of the aphids included in Experiment 1.

### Life table analysis

The raw data of the individual insects (the survivorship, longevity, and female daily fecundity) were analysed according to the age-stage, two-sex life table theory^29^,^30^,^31^ using the computer program TWOSEX-MSChart^50^. The age-stage specific survival rate (*s*_*xj*_), where *x* is age and *j* is the stage; the age-specific survival rate (*l*_*x*_); the age-stage specific fecundity (*f*_*xj*_); and the age-specific fecundity (*m*_*x*_) were determined and used to calculate the net reproductive rate (*R*_0_) as follows:


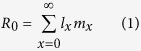


The intrinsic rate of increase (*r*) was estimated by using the iterative bisection method and the Euler-Lotka equation with the age indexed from 0 51:


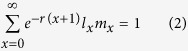


The finite rate (*λ*) was calculated as


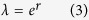


The mean generation time (*T*) was calculated as


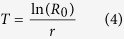


The bootstrap^52^ techniques were used to estimate the variances and standard errors of the population parameters^39^,^40^. To generate less variable results, we used 100,000 replications in bootstrap^39^,^40^. A paired bootstrap test^52^,^53^ was used to compare the differences among treatments using TWOSEX-MSChart^50^.

### Population projection

The growth of *M. dirhodum* population liberated from diseases, predation and parasitism was projected for each treatment based on the life table data using the program TIMING-MSChart^54^ and the approach described in Chi^55^ and Tuan *et al*.^56^.

## Additional Information

**How to cite this article**: Saska, P. *et al*. Treatment by glyphosate-based herbicide alters life history parameters of the rose-grain aphid *Metopolophium dirhodum. Sci. Rep.* **6**, 27801; doi: 10.1038/srep27801 (2016).

## Figures and Tables

**Table 1:** Duration of the development of *Metopolophium dirhodum* as affected by glyphosate-based herbicide application on the parental females.

Stage^a^	Sex^b^	Developmental time in treatments (Mean ± SE)							
		*n*^c^	Control	*n*	Low conc.	*n*	Medium conc.	*n*	High conc.
N1	All	32	2.16 ± 0.08	49	1.98 ± 0.07	49	2.10 ± 0.05	53	2.00 ± 0.05
N2	All	32	2.22 ± 0.11	47	1.98 ± 0.10	39	1.69 ± 0.08	39	2.05 ± 0.12
N3	All	32	2.03 ± 0.10	44	2.02 ± 0.09	36	2.11 ± 0.09	29	1.86 ± 0.14
N4	All	32	2.53 ± 0.13	41	2.59 ± 0.09	31	2.48 ± 0.13	22	2.18 ± 1.14
Adult	All	32	18.12 ± 1.26	41	18.02 ± 1.20	31	15.48 ± 1.22	22	13.77 ± 1.72
N1	F	32	2.16 ± 0.08	41	1.93 ± 0.05	31	2.10 ± 0.05	22	2.05 ± 0.05
N2	F	32	2.22 ± 0.11	41	1.93 ± 0.11	31	1.68 ± 0.10	22	1.95 ± 0.08
N3	F	32	2.03 ± 0.10	41	1.98 ± 0.09	31	2.06 ± 0.09	22	1.91 ± 0.16
N4	F	32	2.53 ± 0.13	41	2.59 ± 0.09	31	2.48 ± 0.13	22	2.18 ± 1.14
Adult	F	32	18.12 ± 1.26	41	18.02 ± 1.20	31	15.48 ± 1.22	22	13.77 ± 1.72
N1	N	–	–	8	2.25 ± 0.31	18	2.11 ± 0.11	31	1.97 ± 0.07
N2	N	–	–	6	2.33 ± 0.21	8	1.75 ± 0.16	17	2.18 ± 0.25
N3	N	–	–	3	2.67 ± 0.33	5	2.40 ± 0.24	7	1.71 ± 0.29
N4	N	–	–	–	–	–	–	–	
Adult	N	–	–	–	–	–	–	–	–

Standard errors (SE) were estimated with bootstrapping (100,000 re-samplings).

^a^N1–N4 indicates the first to fourth instars of the nymphs.

^b^All – all individuals; F – females; N – died before adulthood.

^c^number of individual aphids that completed a stage.

**Table 2:** Reproduction and life table parameters of *Metopolophium dirhodum* as affected by glyphosate-based herbicide application.

Parameter	Glyphosate treatment (Mean ± SE)							
	Control		Low conc.		Medium conc.		High conc.	
Experiment 1 – filial generation								
*λ* (d^−1^)	1.2793 ± 0.0052	a	1.2708 ± 0.0093	a	1.2217 ± 0.0140	b	1.1843 ± 0.019	b
*r* (d^−1^)	0.2463 ± 0.0041	a	0.2397 ± 0.0073	a	0.2002 ± 0.0115	b	0.1692 ± 0.016	b
*R*_0_ (offspring individual^−1^)	41.15 ± 2.60	a	31.30 ± 2.80	b	17.13 ± 2.56	c	10.15 ± 2.03	d
*T* (days)	15.09 ± 0.25	a	14.37 ± 0.21	b	14.19 ± 0.27	b	13.71 ± 0.41	b
Fecundity (laid nymphs female^−1^)	41.16 ± 2.60	a*	38.17 ± 2.31	a*	29.29 ± 2.79	b*	20.09 ± 2.97	b*
Pre-oviposition period (days)	0.78 ± 0.09	ab	0.59 ± 0.09	a	0.87 ± 0.06	b	0.91 ± 0.11	b
Oviposition period (days)	13.91 ± 0.77	ab^NS^	14.80 ± 0.76	a*	11.64 ± 1.05	bc*	9.82 ± 1.09	c^NS^
Pre-adult survival	1.00 ± 0.00	a	0.82 ± 0.05	b	0.59 ± 0.07	c	0.35 ± 0.06	d
Female stage duration (days)	18.13 ± 1.24	a*	18.03 ± 1.18	a*	15.48 ± 1.20	ab*	13.78 ± 1.71	b*
Longevity (days)	27.06 ± 1.27	a	22.92 ± 1.44	b	16.26 ± 1.43	c	10.98 ± 1.19	d
Experiment 2 – treated generation								
Fecundity (laid nymphs female^−1^)	30.17 ± 3.52	a*	19.65 ± 3.88	b*	7.12 ± 2.76	c*	8.25 ± 2.09	c*
Oviposition period (days)	11.77 ± 1.07	a^NS^	8.65 ± 1.54	ab*	4.27 ± 1.45	c*	7.15 ± 1.10	bc^NS^
Female stage duration (days)	13.82 ± 1.44	a*	9.36 ± 1.81	a*	3.52 ± 1.05	b*	5.04 ± 0.97	b*

Standard errors (SE) were estimated with bootstrapping (100,000 re-samplings). The same letter within each row indicates that the groups of treatments are not significantly different from each other based on a paired bootstrap test. *significantly different between Experiments 1 and 2; ^NS^not significantly different between Experiments 1 and 2.
